# Conducting Publishable Research From Special Populations: Studying Children and Non-human Primates With Undergraduate Research Assistants

**DOI:** 10.3389/fpsyg.2019.01030

**Published:** 2019-05-07

**Authors:** Jane B. Childers, Kimberley A. Phillips

**Affiliations:** Department of Psychology, Trinity University, San Antonio, TX, United States

**Keywords:** children, nonhuman primates (NHPs), undergraduate research, laboratory–methods, best practices

Collecting publishable data with only undergraduate research assistants (RAs) is difficult; conducting research with young children or non-human primates (NHPs) adds a layer of difficulty, yet we have been able to successfully sustain and grow research programs in Developmental Psychology and primate Behavioral Neuroscience at Trinity University (TU), a primarily undergraduate institution (PUI) in San Antonio. We each have been conducting research for over 25 years, with most of that time at this type of institution, and have developed effective strategies for publishing articles with undergraduates in this environment.

## Setting Goals

A primary strategy is to set long-term, high-level goals, and work backwards to identify short-term tasks that keep the work focused toward these goals (e.g., Wilkowski and Ferguson, 2016). We subscribe to the “2 weeks, 2 months, 2 years” method, wherein we identify goals (i.e., project completed, manuscript submitted) to be accomplished in 2 years. We then identify tasks to be completed in the next 2 weeks (i.e., literature search) and 2 months (i.e., number of participants run). Involving RAs in this process allows them to see how their contributions contribute to long-term goals. Additionally, we have found that setting up schedules for training and working in the lab within the first week of each semester is a key to getting undergraduates successfully engaged. Undergraduates can be overcommitted, and their schedules fill rapidly. Putting lab times into their schedules early, in writing, is important.

## Recruiting Undergraduate Research Assistants

Recruiting good students and encouraging them to stay in the lab multiple semesters is important to enculturating students into scientific practices (Thiry and Laursen, 2011; Thiry et al., 2012; Linn et al., 2015). Students who move from the “performing without understanding” stage into the “performing with understanding” stage are invaluable team members, who can contribute insights which can lead to co-authorship (Ankrum, 2018). We have developed strategies for identifying students who are likely to be a good match. First, prospective RAs need to have some specific background knowledge, such as successful completion of an introductory course in neuroscience (KAP) or other coursework in Psychology (JC). Students submit a written application, which includes questions pertaining to coursework, motivation, work ethic, and ethical considerations when working with special populations. We then talk informally with other faculty and interview students before accepting them. Prior experience engaging with young children or working with animals helps students more quickly make meaningful contributions to research projects. We seek out students who can work independently and collaboratively, and can enrich our labs with diverse backgrounds and perspectives. We have found that a student's drive and passion for research are typically better predictors of success than is GPA, though maintaining a minimum GPA is required.

We usually have more students interested in being in the lab than positions, and previously have accepted additional students which resulted in a larger lab group than we found to be manageable (<12 students/semester). As a consequence, the productivity of the lab actually slowed. Thus, we now only accept a set number of students (typically 5 or less for KAP; 8–10 for JC). Returning students have priority over new students, as long as they have shown productivity.

## Additional Challenges when Conducting Studies Off Campus

Our data collection is largely accomplished off campus at a National Primate Research Center (KAP) or at local child care centers (JC). Thus, two hurdles must be addressed early: planning transportation to and from sites, and completing the background and security checks needed for working with these special populations. Transportation can be a barrier, particularly for students of underrepresented groups. We coordinate student schedules so that they can carpool to the research sites, which incurs the additional benefit of ensuring students are collecting data in pairs, increasing fidelity to protocols. (For liability reasons, we do not have students carpool with us.) In addition, an Office of Risk Management aids with the various legal forms that must be completed and filed, including fingerprint background checks. Some University support is helpful, as a fingerprint background check is costly (~$40). Students working at the National Primate Research Center must undergo additional security clearances and medical screenings, which can take up to 2 months. By planning for these obstacles, we can quickly incorporate new RAs into our research teams.

## Designing Research Procedures for Undergraduate RAs

**Figure 1:**
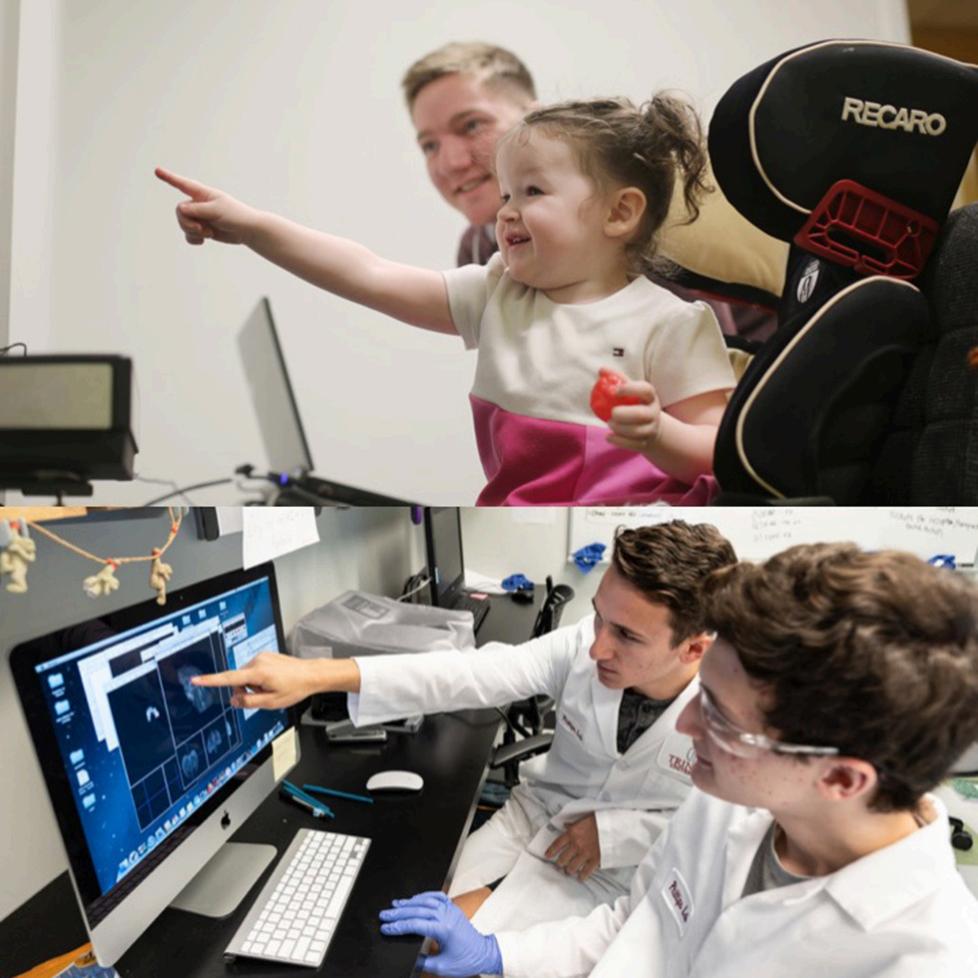
Undergraduate Research assistant collecting data from a child using an eye-tracker **(top panel)**; undergraduates discussing analysis of MRI scan **(bottom panel)**. Written informed consent was obtained from the depicted adults and students, and the parents of the depicted child, for the publication of this image. All appropriate permissions were obtained from the University/copyright holders for the use of this image in the manuscript. Photo credit: Trinity University.

Another key consideration is designing studies that can be conducted with undergraduate RAs (see Figure 1). While we have been fortunate to continue our research programs with children and nonhuman primates (NHPs), we have modified our research agendas for success at a PUI. First, procedures must be tailored to the participants available in the environment. For JC, this meant not conducting research with infants (who often need to be brought to campus) but focusing on preschool-aged children (who can be recruited in child care centers). KAP utilizes the resources of a National Primate Research Center (at which a veterinarian and other technical staff are available) rather than house animals at an on-campus vivarium.

The procedures we use in our research include some that are highly technical–including eye tracking and magnetic resonance imaging (MRI). We find our RAs to be quite capable of understanding and successfully working with, and analyzing data from, these techniques. We work with our students to develop written protocols for these procedures. Repeating students can be given the responsibility of training new students in person on each procedure, with the PI in attendance to be sure training is accurate. Students create their own training videos (with supervision), to help remind new RAs of procedures after training. These activities help continuing students take ownership of projects, develop a deeper investment in research, and foster leadership skills.

JC's team typically conducts multiple studies simultaneously, with some of these studies including procedures that are easier to use (e.g., behavioral enactment and studies that use iPads), and some studies including procedures that are more technical (i.e., using an eye tracker which uses near-infrared light and corneal reflectance to track where in a dynamic event a child is visually attending; funded through an NIH R15 grant). Using a more technical procedure has its costs in terms of potential data loss due to equipment issues, but at the same time, provides students with experiences that can give students an edge when applying to positions after college. These procedures allow students the chance to assist with stimuli creation (e.g., filming and editing video; creating “live” events), which also enriches their experience. A difficulty in working with 2- to 4-year-olds is that participants are not always engaged or compliant in obvious ways. To help students prepare for this, new students code videos of prior experimental sessions to see how other students have handled different situations (and to provide interrater reliability coding). We also pair a new student with a returning student who can mentor them, especially when they are collecting data off campus.

KAP integrates behavioral or cognitive data with analyses of brain structure and function obtained from neuroimaging. She utilizes the research imaging scanners at the local medical school for acquisition of structural MRI, resting-state functional MRI, and diffusion tensor imaging. She works closely with MRI physicists to develop the scanning protocols and veterinary staff to care for the animals. Students collect the behavioral or cognitive data (e.g., motor learning) from NHPs and assist with the acquisition of the scans. Although analysis and interpretation of neuroimaging can be challenging for students, many are eager for the opportunity and devote considerable time to the task. Such work often requires troubleshooting that involves editing Unix code, as the brain image analysis software was developed for humans. In addition to brain scans, KAP also uses some behavioral protocols (e.g., testing the effects of exercise on cognition; primate problem solving) that RAs can help develop and administer.

As undergraduate RAs collect data, systems must be in place to monitor their progress and be sure students are not engaging in “experimenter drift.” We use multiple coders of behavioral data, have students submit weekly lab reports (short emails of progress and challenges), and provide opportunities for students to write. Often, papers are part of our Supervised Research course, which gives students course credit for research, provides faculty with a course that can count toward their teaching load, and gives students practice at delivering oral presentations as well as writing up projects. These papers can be helpful because they provide a record of where each project was in a semester—which can be useful as projects evolve. Additionally, a poster can be presented at the end of the summer research session, and students are given chances to present posters (or rarely papers) at major national and international conferences. Inviting RAs who have contributed meaningfully to the research over multiple semesters to write portions of conference abstracts encourages students to continue in the lab. It also serves as great experience as they later create a research poster that they often have a chance to present at a conference. At times, strong students are encouraged to complete an undergraduate thesis which stems from research in the lab. Theses take three semesters so that students have ample time to design and implement the study (S1 and S2), and have time to write (S2 and S3). Multiple drafts are submitted for revision in stages. These thesis papers have been great starts to papers that can later be submitted for publication with student(s) as a co-author (Childers et al., 2012, 2014, 2016; Phillips et al., 2018;Phillips et al., 2019).

## Financial Support

Research is expensive. Our institution helps us fund our highly productive undergraduate-centered research programs by providing some departmental support for research and by employing a Sponsored Research Officer to assist with applying for external support. We have been successful in applying for Federal grants, including the NIH R15 and NSF REU grant programs. We have also found that connecting with local Foundations for possible funding can be useful; initiating and sustaining these relationships may yield not only funding but also opportunities for collaboration.

We know our undergraduate RAs are juggling courses, work, and time in the lab. One study reported 70–80% of college students are also in the labor market (Carnevale et al., 2015). If students enroll in 15 credit hours/semester, and study 3 h for every 1 h in class, students are spending approximately 45 h/week with class-related activities. We have found that providing students with support can ensure a minimum number of hours are spent in the lab. Many students during the school year and summer receive course credit. Some top students can be funded through internal funding or grants during the summer, which they must apply for– and this helps them to start thinking about research and promotes writing.

## Conclusions

In summary, even at PUIs, it is possible to conduct high quality, publishable research with undergraduate RAs. Building the capacity of undergraduate RAs to contribute to the success of the lab has been instrumental in allowing us to regularly publish our findings.

## Author Contributions

All authors listed have made a substantial, direct and intellectual contribution to the work, and approved it for publication.

### Conflict of Interest Statement

The authors declare that the research was conducted in the absence of any commercial or financial relationships that could be construed as a potential conflict of interest.
